# Supplementary material for: Molecular classification of the placebo effect in nausea
Source: PLoS One. 2020 Sep 23;15(9):e0238533. doi: 10.1371/journal.pone.0238533 (PMC7511022; doi:10.1371/journal.pone.0238533)
Supplement: S1 Table — (PDF) [file pone.0238533.s003.pdf]

S1 Table: ANCOVA results for DAS-Nausea, DAS-MS, and DAS-NTT.

Measure	Control group			Placebo group			2-way ANOVA		
	n	*mean*	*s.d.*	*n*	*mean*	*s.d.*	*group*	*sex*	*group by sex*
DAS-Nausea (NRS 0-10)							$F_{(1,86)}$ = 44.83, P < 0.001	$F_{(1,86)}$ = 3.05, P = 0.084	$F_{(1,86)}$ = 1.01, P = 0.318
female	15	-0.32	1.33	30	-3.10	1.63			
male	15	-1.31	1.64	30	-3.37	1.71			
total	30	-0.82	1.55	60	-3.23	1.66			
DAS-MS							$F_{(1,84)}$ = 14.93, P < 0.001	$F_{(1,84)}$ = 0.21, P = 0.648	$F_{(1,84)}$ = 0.47, P = 0.493
female	14	-0.10	0.34	29	-0.53	0.44			
male	15	-0.12	0.38	30	-0.42	0.43			
total	29	-0.11	0.36	59	-0.48	0.44			
DAS-NTT (log ratio)							$F_{(1,83)}$ = 0.51, P = 0.476	$F_{(1,83)}$ = 0.0, P = 0.996	$F_{(1,83)}$ = 4.16, P = 0.044
female	13	-0.18	1.41	29	0.82	1.51			
male	15	0.56	1.69	30	0.08	1.65			
total	28	0.22	1.58	59	0.44	1.61			

Abbreviations: DAS, day-adjusted score; NRS, numeric rating scale; bpm, beats per minute; MS, motion sickness; NTT, normo-to-tachy ratio
