# Supplementary material for: Molecular classification of the placebo effect in nausea
Source: PLoS One. 2020 Sep 23;15(9):e0238533. doi: 10.1371/journal.pone.0238533 (PMC7511022; doi:10.1371/journal.pone.0238533)
Supplement: S2 Table — (PDF) [file pone.0238533.s004.pdf]

S2 Table: Proteins for which a significant amount of variance could be explained by 'group', 'sex', 'DAS-Nausea', or by any of the interaction terms.

Gene Names	Protein Accessions	Intercept	Nausea	Group	Sex	Nausea X Group	Nau-sea X Sex	Group X Sex	Nausea X Group X Sex
**ACSL3**	O95573	0.003	**0.000**	**0.011**	**0.001**	**0.000**	**0.001**	**0.013**	**0.000**
**ACTN2**	P35609	0.003	**0.010**	**0.009**	**0.006**	**0.002**	**0.016**	**0.022**	**0.003**
**ADAD1**	Q96M93	0.436	0.518	0.589	0.177	0.565	**0.026**	0.666	**0.004**
**ALB;APOH; APOA2**	P02768;P02749; P02652	0.481	0.361	0.486	0.147	0.061	**0.027**	0.099	**0.004**
**ALG2**	Q9H553	0.275	0.154	0.305	0.184	0.247	**0.003**	0.254	**0.004**
**ANKRD11**	Q6UB99	0.297	0.785	0.307	**0.040**	0.395	0.067	0.072	**0.006**
**ANXA2**	P07355	0.834	0.978	0.853	0.458	**0.042**	0.327	0.887	**0.007**
**AOPEP**	Q8N6M6	0.145	**0.000**	0.443	0.324	**0.000**	**0.001**	0.527	**0.007**
**APOA2**	P02652	0.266	0.487	0.255	0.058	0.457	**0.010**	0.091	**0.009**
**APOB**	P04114	0.428	0.531	0.477	0.138	0.368	0.068	0.456	**0.012**
**APOL1**	O14791	0.151	0.582	0.176	0.069	0.174	0.114	0.056	**0.012**
**APP**	P05067	0.034	0.113	**0.030**	**0.041**	**0.049**	0.060	**0.036**	**0.012**
**AZGP1**	P25311	0.015	**0.006**	**0.007**	**0.003**	**0.024**	**0.006**	**0.008**	**0.015**
**C1S;MMRN1;C3; PLEC;C1R**	P09871;Q13201; P01024;Q15149; P04208;P00736	0.000	**0.000**	**0.000**	**0.000**	**0.002**	**0.002**	**0.001**	**0.015**
**C3;ACTB; GPATCH4; ACTBL2;ACTC1**	P01701;P01024; P60709;Q5T3I0; Q562R1;P68032	0.001	**0.000**	**0.003**	**0.003**	**0.000**	**0.000**	**0.019**	**0.000**
**C4BPA**	P04003	0.114	0.075	0.268	**0.041**	0.065	**0.035**	0.149	**0.015**
**C4BPA;A1BG; C1QA;APOB**	P04003;P04217; P02745;P04114	0.278	0.454	0.328	0.115	0.150	0.123	0.116	**0.016**
**CARD9**	Q9H257	0.950	0.902	0.921	0.064	0.981	**0.025**	0.084	**0.016**
**CCDC160**	A6NGH7	0.271	0.528	0.390	0.124	0.209	**0.018**	0.214	**0.017**
**CDC6;IGHM; DSP;EZR;**	P04220;Q99741; P01871;P15924; P15311;P01768	0.000	**0.000**	**0.000**	**0.000**	**0.000**	**0.000**	**0.000**	**0.000**
**CFH**	P08603	0.477	0.748	0.530	0.201	0.325	0.110	0.134	**0.019**
**CFHR4**	Q92496	0.005	**0.001**	**0.024**	**0.017**	**0.001**	**0.028**	0.077	**0.024**
**COL6A5**	A8TX70	0.208	0.703	0.219	0.074	0.344	0.256	0.061	**0.025**
**DDX42**	Q86XP3	0.396	0.630	0.368	0.221	0.237	0.191	0.417	**0.025**
**DMRTA1**	Q5VZB9	0.508	0.744	0.628	0.775	0.788	0.799	0.792	**0.026**
**DNHD1**	Q96M86	0.270	0.670	0.319	0.086	0.341	0.099	0.072	**0.026**
**EDEM3**	Q9BZQ6	0.090	0.140	0.053	0.094	**0.021**	0.120	**0.038**	**0.026**
**EHMT1**	Q9H9B1	0.622	0.898	0.832	0.258	0.670	0.218	0.490	**0.028**
**EIF6**	P56537	0.478	0.391	0.660	0.395	0.182	0.173	0.513	**0.028**
**EPM2AIP1**	Q7L775	0.933	0.904	0.899	0.054	0.939	**0.027**	0.082	**0.029**
**FCGR3B**	O75015	0.340	0.431	0.315	0.224	0.424	**0.026**	0.221	**0.031**
**FER1L5**	A0AVI2	0.649	0.865	0.955	0.228	0.336	0.155	0.430	**0.031**
**FGFRL1**	Q8N441	0.184	0.522	0.195	0.128	0.133	0.183	0.092	**0.032**
**FLG**	P20930	0.184	0.522	0.195	0.128	0.133	0.183	0.092	**0.032**
**FLNA**	P21333	0.945	0.986	0.761	0.481	0.358	0.393	0.791	**0.033**
**FN1**	P02751	0.104	0.185	**0.037**	**0.034**	0.225	**0.047**	**0.007**	**0.034**

Gene Names	Protein Accessions	Intercept	Nausea	Group	Sex	Nausea X Group	Nau-sea X Sex	Group X Sex	Nausea X Group X Sex
**FZD3**	Q9NPG1	0.061	0.102	0.125	**0.042**	**0.040**	0.058	0.115	**0.034**
**GP1BA**	P07359	0.006	0.094	**0.020**	**0.011**	**0.017**	0.124	**0.039**	**0.037**
**GPR108**	Q9NPR9	0.313	0.183	0.352	0.149	0.122	**0.027**	0.177	**0.037**
**GRHL2**	Q6ISB3	0.138	0.259	0.197	**0.033**	0.120	**0.047**	**0.045**	**0.038**
**GRIK4**	Q16099	0.092	0.179	0.062	0.069	0.154	**0.036**	0.056	**0.039**
**HEPHL1**	Q6MZM0	0.501	0.953	0.780	0.194	0.554	0.194	0.264	**0.039**
**HNRNPA2B1**	P22626	0.478	0.919	0.711	0.251	0.908	0.241	0.293	**0.039**
**HSPB1**	P04792	0.035	0.059	**0.048**	0.098	0.053	0.058	0.210	**0.041**
**IGFALS**	P35858	0.476	0.823	0.564	0.225	0.586	0.134	0.307	**0.041**
**IGHG4;ALB; MCF2L2;IGHM; FGG;CLTC**	P04220;P01861; P02768;Q86YR7; P01871;P02679; Q00610	0.000	**0.000**	**0.000**	**0.000**	**0.000**	**0.000**	**0.001**	**0.000**
**JAKMIP1**	Q96N16	0.152	0.181	0.089	**0.011**	0.191	**0.024**	**0.014**	**0.043**
**KBTBD4**	Q9NVX7	0.346	0.525	0.288	0.649	0.663	0.314	0.763	**0.043**
**KRT13**	P13646	0.381	0.614	0.325	0.537	0.489	0.708	0.583	**0.046**
**KRT16**	P08779	0.261	0.617	0.258	0.168	0.331	0.222	0.186	**0.048**
**KRT6B**	P04259	0.829	0.973	0.702	0.577	0.865	**0.018**	0.367	**0.048**
**KRT84**	Q9NSB2	0.217	0.511	0.383	0.104	0.217	0.170	0.081	**0.048**
**LRP11**	Q86VZ4	0.390	**0.043**	0.535	0.641	0.062	0.290	0.918	0.053
**MAPK8IP2**	Q13387	0.109	0.231	0.149	**0.024**	0.179	0.078	**0.047**	0.053
**MED30**	Q96HR3	0.025	0.078	**0.015**	**0.019**	0.099	**0.043**	**0.016**	0.063
**MYO5B**	Q9ULV0	0.034	**0.024**	**0.047**	**0.033**	0.057	**0.016**	0.051	0.065
**NKIRAS1**	Q9NYS0	0.401	**0.040**	0.876	0.444	0.124	0.054	0.948	0.075
**NRXN1**	Q9ULB1	0.026	0.076	**0.012**	**0.015**	0.123	0.058	**0.005**	0.096
**OSBPL1A**	Q9BXW6	0.141	**0.048**	0.379	0.268	**0.040**	0.249	0.729	0.104
**PHACTR1**	Q9C0D0	0.073	**0.019**	0.079	0.058	0.100	**0.037**	0.059	0.116
**PHLDA1**	Q8WV24	0.007	0.062	**0.027**	**0.023**	0.068	0.121	0.056	0.128
**PKP1**	Q13835	0.112	0.218	**0.026**	0.336	0.241	0.286	0.175	0.146
**PLOD2**	O00469	0.116	0.203	0.077	**0.035**	0.173	**0.045**	**0.009**	0.148
**POLI**	Q9UNA4	0.030	0.104	0.053	**0.041**	0.112	**0.035**	0.090	0.156
**POMT2**	Q9UKY4	0.162	0.276	0.101	0.113	0.362	0.208	**0.049**	0.192
**PON1**	P27169	0.583	0.327	0.567	0.514	**0.041**	0.265	0.210	0.193
**POSTN**	Q15063	0.626	0.126	0.885	0.770	0.404	**0.044**	0.867	0.211
**RBM44**	Q6ZP01	0.043	0.247	**0.045**	**0.009**	0.197	0.152	**0.012**	0.233
**SBSN**	Q6UWP8	0.011	**0.044**	**0.004**	**0.024**	0.589	0.412	**0.005**	0.247
**SCN2A**	Q99250	0.106	**0.032**	0.128	0.220	0.071	0.128	0.345	0.250
**SERPINC1**	P01008	0.089	0.148	**0.043**	0.298	0.478	0.687	**0.042**	0.267
**SERPINF1**	P36955	0.042	0.087	**0.021**	0.056	0.451	**0.032**	**0.048**	0.306
**SGSM1**	Q2NKQ1	0.193	**0.049**	0.375	0.831	0.158	0.257	0.444	0.316
**SKAP1**	Q86WV1	0.018	**0.023**	**0.016**	**0.045**	0.284	**0.030**	0.057	0.344
**SLC16A8**	O95907	0.119	**0.032**	0.074	0.448	0.133	0.269	0.306	0.350
**SLFN11**	Q7Z7L1	0.020	0.150	**0.005**	**0.041**	0.419	0.145	**0.024**	0.354
**SOS2**	Q07890	0.234	0.585	0.170	0.120	0.389	0.159	**0.029**	0.354
**TEX11**	Q8IYF3	0.161	0.112	0.118	0.139	0.204	0.094	**0.031**	0.376

Gene Names	Protein Accessions	Intercept	Nausea	Group	Sex	Nausea X Group	Nau-sea X Sex	Group X Sex	Nausea X Group X Sex
**TSC1**	Q92574	0.080	0.050	**0.047**	0.246	0.365	0.075	0.269	0.420
**TTK**	P33981	0.180	**0.047**	0.230	0.456	0.083	0.435	0.619	0.421
**ZG16B**	Q96DA0	0.014	0.057	**0.012**	**0.025**	0.139	0.159	0.075	0.488
	P01611	0.043	0.242	**0.033**	0.072	0.244	0.488	**0.021**	0.548
	P01762	0.045	**0.018**	0.070	0.164	**0.037**	0.715	0.168	0.564
	P01768	0.109	0.445	**0.049**	0.140	0.891	0.906	0.060	0.632
	P04211	0.106	0.323	0.063	0.060	0.340	0.372	**0.038**	0.649
	P01707	0.161	0.135	0.121	0.073	0.164	0.214	**0.033**	0.653
	A8MT66	0.102	**0.036**	0.093	0.246	0.086	0.389	0.120	0.906
	P04430	0.080	0.601	**0.046**	0.207	0.856	0.559	0.232	0.906
	P80422	0.328	0.487	0.311	0.155	0.406	0.680	**0.033**	0.926

Abbreviations: DAS, day-adjusted scores.
