# Supplementary material for: Molecular classification of the placebo effect in nausea
Source: PLoS One. 2020 Sep 23;15(9):e0238533. doi: 10.1371/journal.pone.0238533 (PMC7511022; doi:10.1371/journal.pone.0238533)
Supplement: S3 Table — (PDF) [file pone.0238533.s005.pdf]

S3 Table: Proteins for which a significant amount of variance could be explained by 'group', 'sex', 'DAS-MS', or by any of the interaction terms.

Gene Names	Protein Accessions	Intercept	MS	Group	sex	MS X Group	MS X sex	Group X sex	MS X Group X sex
					P-value				
**A1BG**	P04217	0.035	**0.000**	0.147	0.097	**0.000**	**0.001**	0.156	**0.001**
**ABCA13**	Q86UQ4	0.562	**0.000**	0.490	0.624	**0.000**	**0.000**	0.674	**0.001**
**ADAD1**	Q96M93	0.159	**0.001**	0.629	0.057	**0.014**	**0.000**	0.275	**0.002**
**ALB;**	P02768;P01619	0.208	**0.011**	0.308	0.887	**0.005**	**0.022**	0.767	**0.006**
**ALB;C3;APOB**	P01765;P02768;P01024; P04114	0.037	**0.000**	0.227	0.195	**0.000**	**0.001**	0.746	**0.000**
**ALB;FGA;LYZ; VWF;LOXHD1;**	P02768;P02671;P61626; P04275;Q8IVV2;P04208	0.052	**0.000**	0.051	0.088	**0.001**	**0.003**	0.069	**0.012**
**ALOX12B**	O75342	0.837	0.442	0.969	0.859	0.211	0.207	0.870	**0.013**
**ANXA2**	P07355	0.399	0.778	0.848	0.156	0.388	0.160	0.665	**0.017**
**ARHGAP10**	A1A4S6	0.035	**0.000**	**0.016**	**0.020**	**0.001**	**0.005**	**0.048**	**0.017**
**ATR**	Q13535	0.836	**0.015**	0.663	0.931	**0.013**	**0.030**	0.783	**0.024**
**AZGP1**	P25311	0.182	**0.033**	0.185	0.188	0.053	**0.040**	0.349	**0.025**
**BIN2**	Q9UBW5	0.062	0.112	0.152	**0.014**	**0.049**	0.122	0.106	**0.035**
**BLMH**	Q13867	0.103	0.112	0.162	0.073	0.313	**0.044**	0.210	**0.037**
**BRMS1**	Q9HCU9	0.020	**0.006**	0.147	0.067	**0.013**	**0.023**	0.203	**0.037**
**CAB39L**	Q9H9S4	0.713	**0.001**	0.903	0.440	**0.004**	**0.013**	0.414	**0.043**
**CCDC160**	A6NGH7	0.052	**0.013**	0.070	0.148	**0.021**	0.062	0.180	**0.044**
**CDC6;IGHM;DSP; EZR;**	P04220;Q99741;P01871; P15924;P15311;P01768	0.003	**0.021**	**0.003**	**0.003**	0.076	**0.000**	**0.006**	**0.000**
**CEP290**	O15078	0.034	**0.010**	0.058	**0.049**	**0.026**	**0.029**	0.066	**0.045**
**CFD**	P00746	0.239	0.476	0.068	0.303	0.567	**0.016**	0.053	**0.045**
**CLEC11A**	Q9Y240	0.056	0.083	**0.048**	**0.007**	**0.027**	0.062	**0.001**	**0.048**
**COLEC10**	Q9Y6Z7	0.298	**0.020**	0.544	0.535	**0.033**	0.054	0.448	0.051
**CREBRF**	Q8IUR6	0.176	0.625	0.182	**0.042**	0.445	0.225	0.069	0.056
**DDB1**	Q16531	0.750	**0.050**	0.932	0.860	0.101	**0.037**	0.776	0.059
**DDX54**	Q8TDD1	0.063	**0.006**	0.159	0.187	**0.003**	0.050	0.161	0.060
**DNHD1**	Q96M86	0.828	0.066	0.737	0.584	0.104	**0.027**	0.402	0.065
**ENO1**	P06733	0.950	0.080	0.168	0.963	**0.026**	0.127	0.122	0.066
**EPM2AIP1**	Q7L775	0.100	0.161	**0.021**	0.179	0.164	0.160	0.072	0.066
**FBLN5**	Q9UBX5	0.828	0.927	0.698	0.260	0.834	**0.020**	0.320	0.073
**FSTL1**	Q12841	0.001	**0.013**	**0.014**	**0.018**	**0.026**	**0.046**	0.059	0.075
**FZD3**	Q9NPG1	0.019	0.114	0.105	0.208	**0.014**	0.226	0.657	0.083
**GRXCR2**	A6NFK2	0.577	0.129	0.664	0.842	**0.044**	0.203	0.451	0.084
**GSN**	P06396	0.404	0.060	0.421	0.137	**0.047**	0.111	0.055	0.084
**GUCY1B2**	O75343	0.014	**0.025**	0.076	0.085	**0.030**	0.096	0.337	0.095
**GYLTL1B**	Q8N3Y3	0.211	0.095	0.283	0.668	**0.019**	0.226	0.483	0.100
**HPS3**	Q969F9	0.479	**0.008**	0.722	0.776	**0.035**	**0.043**	0.794	0.109
**HRG**	P04196	0.060	**0.024**	0.118	0.200	**0.019**	0.096	0.331	0.119
**HSD3B1**	P14060	0.067	**0.003**	0.066	0.867	**0.007**	0.124	0.755	0.125
**HTT**	P42858	0.379	**0.023**	0.291	0.452	0.083	**0.010**	0.260	0.127
**IGFALS**	P35858	0.949	0.111	0.797	0.854	0.274	**0.040**	0.958	0.128

Gene Names	Protein Accessions	Intercept	MS	Group	sex	MS X Group	MS X sex	Group X sex	MS X Group X sex
**IGHG2**	P01859	0.766	0.105	0.416	0.230	**0.037**	0.680	0.132	0.130
**IGHM;**	P01871;P01773	0.909	0.703	0.531	0.073	0.667	0.083	**0.022**	0.139
**IZUMO4**	Q1ZYL8	0.088	**0.031**	0.073	0.061	**0.014**	0.123	**0.022**	0.142
**KIF14**	Q15058	0.144	**0.031**	0.138	0.515	**0.009**	0.117	0.253	0.143
**KIF17**	Q9P2E2	0.109	0.056	0.334	0.219	**0.049**	0.341	0.422	0.146
**KRT10**	P13645	0.900	**0.041**	0.337	0.955	0.100	**0.034**	0.341	0.153
**KRT13**	P13646	0.903	0.955	0.983	**0.047**	0.909	0.131	0.152	0.209
**KRT6B**	P04259	0.281	0.587	0.243	0.063	0.590	0.274	**0.015**	0.225
**KRT9**	P35527	0.853	0.453	0.297	0.429	0.886	0.082	**0.047**	0.226
**MAPT**	P10636	0.215	0.094	0.785	**0.004**	0.567	**0.035**	0.103	0.234
**MYO5B**	Q9ULV0	0.088	0.278	0.103	**0.048**	0.220	0.337	0.051	0.235
**NDUFS2**	O75306	0.085	0.287	**0.034**	0.090	0.402	0.254	**0.030**	0.252
**NRIP1**	P48552	0.647	**0.050**	0.723	0.723	**0.025**	0.251	0.358	0.253
**NRXN1**	Q9ULB1	0.484	0.488	0.288	0.063	0.281	0.270	**0.020**	0.254
**OSBPL1A**	Q9BXW6	0.895	**0.036**	0.987	0.674	**0.030**	0.198	0.928	0.257
**OSBPL5**	Q9H0X9	0.695	**0.038**	0.272	0.470	0.232	0.171	0.955	0.266
**PHACTR1**	Q9C0D0	0.309	0.220	0.242	0.119	0.571	0.118	**0.020**	0.275
**PLCXD1**	Q9NUJ7	0.119	**0.017**	0.371	0.178	0.055	**0.048**	0.312	0.288
**PLG**	P00747	0.377	**0.041**	0.621	0.512	**0.037**	0.475	0.571	0.301
**POLI**	Q9UNA4	0.131	0.173	**0.049**	0.513	0.117	0.618	0.220	0.324
**PPIP5K2**	O43314	0.116	0.222	**0.015**	0.473	0.288	0.266	0.149	0.334
**PPP4R3CP**	Q6ZMV5	0.349	**0.014**	0.238	0.693	0.081	0.059	0.296	0.334
**RICTOR**	Q6R327	0.065	0.967	**0.045**	**0.040**	0.835	0.871	**0.012**	0.336
**RNH1**	P13489	0.062	0.498	0.084	0.111	0.273	0.682	**0.042**	0.391
**S100A7**	P31151	0.427	0.263	0.180	0.263	0.300	0.324	**0.047**	0.397
**S100A9**	P06702	0.019	0.096	**0.048**	**0.037**	0.139	0.372	0.069	0.454
**SERPINB12**	Q96P63	0.864	**0.038**	0.777	0.568	0.089	0.245	0.515	0.462
**SERPINF1**	P36955	0.149	0.671	0.112	0.097	0.576	0.704	**0.032**	0.503
**SGSM1**	Q2NKQ1	0.102	0.167	0.066	0.100	0.162	0.375	**0.039**	0.513
**SHANK1**	Q9Y566	0.240	**0.018**	0.191	0.873	**0.022**	0.241	0.409	0.513
**SKAP1**	Q86WV1	0.691	0.697	0.779	0.054	0.723	0.175	**0.020**	0.548
**SLC35C2**	Q9NQQ7	0.129	**0.036**	0.153	0.742	0.200	0.206	0.861	0.600
**SORCS2**	Q96PQ0	0.599	0.435	0.649	0.098	0.638	0.376	**0.037**	0.645
**SPEN**	Q96T58	0.140	0.817	0.343	**0.042**	0.840	0.624	0.204	0.681
**SPP1**	P10451	0.358	0.054	0.376	0.618	**0.033**	0.343	0.363	0.724
**SVOPL**	Q8N434	0.137	**0.030**	0.094	0.788	0.167	0.293	0.778	0.749
**SYNE1**	Q8NF91	0.874	**0.009**	0.500	0.164	0.056	0.217	0.075	0.764
**TRAP1**	Q12931	0.970	0.901	0.777	0.342	0.955	**0.046**	0.626	0.772
**TXN**	P10599	0.193	0.915	0.388	**0.044**	0.857	0.552	0.071	0.794
**VCAM1**	P19320	0.307	**0.018**	0.264	0.950	0.331	0.084	0.959	0.835
**ZG16B**	Q96DA0	0.447	0.752	0.229	0.174	0.996	0.874	**0.025**	0.856
**ZNF438**	Q7Z4V0	0.564	**0.021**	0.316	0.484	0.086	0.465	0.906	0.866
	P01596	0.058	0.901	**0.007**	0.256	0.982	0.982	**0.036**	0.868
	P01762	0.056	0.087	**0.027**	0.353	0.437	0.321	0.317	0.924

Gene Names	Protein Accessions	Intercept	MS	Group	sex	MS X Group	MS X sex	Group X sex	MS X Group X sex
	P80422	0.028	0.119	**0.008**	0.384	0.300	0.508	0.171	0.982
	P01707	0.211	0.789	0.127	0.151	0.952	0.388	**0.028**	0.996

Abbreviations: DAS-MS, day-adjusted scores of motion sickness.
