# Supplementary material for: Molecular classification of the placebo effect in nausea
Source: PLoS One. 2020 Sep 23;15(9):e0238533. doi: 10.1371/journal.pone.0238533 (PMC7511022; doi:10.1371/journal.pone.0238533)
Supplement: S4 Table — (PDF) [file pone.0238533.s006.pdf]

**S4 Table: Proteins for which a significant amount of variance could be explained by 'group', 'sex', 'DAS-NTT', or by any of the interaction terms.**

| Gene Names | PG Protein Accessions | Intercept | NTT | Group | sex | NTT X Group | NTT X Sex | Group X Sex | NTT X Group X sex |
|---|---|---|---|---|---|---|---|---|---|
| | | *P*-Value | | | | | | | |
| **ABCA13** | Q86UQ4 | 0.810 | 0.497 | 0.677 | 0.053 | 0.694 | 0.051 | **0.013** | 0.704 |
| **ABCA6** | Q8N139 | 0.439 | 0.242 | **0.015** | 0.533 | 0.994 | 0.270 | **0.048** | 0.984 |
| **ABCC9** | O60706 | 0.045 | 0.191 | **0.037** | 0.329 | 0.181 | **0.044** | 0.807 | **0.013** |
| **ACTN2** | P35609 | 0.018 | 0.220 | 0.076 | **0.012** | 0.197 | 0.444 | **0.029** | 0.132 |
| **ALB;VNN1; FGG; TECTA** | P02768;O95497;P02679; P01597;O75443 | 0.255 | 0.951 | **0.019** | 0.748 | 0.311 | 0.876 | 0.180 | 0.208 |
| **ANKRD11** | Q6UB99 | 0.898 | **0.017** | 0.934 | 0.601 | **0.040** | 0.124 | 0.553 | 0.391 |
| **ANKRD55** | Q3KP44 | 0.002 | 0.225 | **0.002** | **0.002** | 0.436 | 0.413 | **0.007** | 0.456 |
| **APOC4** | P55056 | 0.011 | **0.024** | **0.032** | **0.004** | 0.189 | 0.196 | **0.015** | 0.555 |
| **APOM** | O95445 | 0.213 | 0.713 | 0.378 | 0.238 | 0.769 | 0.573 | **0.044** | 0.347 |
| **APP** | P05067 | 0.025 | 0.927 | **0.008** | **0.009** | 0.444 | 0.905 | **0.007** | 0.677 |
| **ATAD2** | Q6PL18 | 0.276 | **0.041** | 0.263 | 0.758 | 0.157 | 0.123 | 0.612 | 0.116 |
| **AZGP1** | P25311 | 0.038 | 0.621 | 0.064 | 0.071 | 0.640 | 0.621 | **0.023** | 0.540 |
| **B3GNT4** | Q9C0J1 | 0.490 | **0.045** | 0.339 | 0.550 | **0.037** | **0.016** | 0.223 | **0.040** |
| **BIN2** | Q9UBW5 | 0.033 | 0.100 | **0.045** | 0.232 | 0.639 | 0.258 | 0.475 | 0.998 |
| **BLMH** | Q13867 | 0.128 | 0.808 | 0.237 | **0.042** | 0.951 | 0.783 | 0.162 | 0.463 |
| **C5;APOA4** | P01031;P01766; P06727 | 0.050 | **0.009** | 0.265 | **0.021** | **0.002** | 0.057 | 0.069 | 0.051 |
| **C5orf42** | Q9H799 | 0.092 | 0.246 | 0.085 | 0.064 | 0.326 | 0.496 | **0.044** | 0.819 |
| **CAB39L** | Q9H9S4 | 0.084 | 0.610 | 0.102 | **0.046** | 0.715 | 0.658 | **0.044** | 0.659 |
| **CD44** | P16070 | 0.732 | 0.511 | 0.963 | 0.989 | 0.549 | **0.033** | 0.818 | 0.151 |
| **CFHR2** | P36980 | 0.071 | 0.406 | **0.026** | 0.113 | 0.950 | 0.405 | **0.042** | 0.909 |
| **CNTNAP4** | Q9C0A0 | 0.331 | 0.071 | 0.295 | 0.728 | 0.201 | **0.006** | 0.659 | **0.022** |
| **COL1A2** | P08123;P01619 | 0.992 | 0.239 | 0.967 | 0.604 | 0.073 | 0.196 | 0.520 | **0.045** |
| **COL6A3** | P12111 | 0.954 | 0.080 | 0.639 | 0.431 | 0.052 | 0.138 | 0.150 | **0.024** |
| **CP** | P00450 | 0.106 | 0.696 | 0.096 | 0.275 | 0.927 | 0.744 | **0.040** | 0.795 |
| **CPA4** | Q9UI42 | 0.696 | 0.838 | 0.986 | **0.042** | 0.473 | 0.465 | 0.095 | 0.219 |
| **CPN2** | P22792 | 0.310 | 0.088 | 0.899 | 0.381 | **0.025** | 0.097 | 0.907 | 0.082 |
| **DAAM1** | Q9Y4D1 | 0.106 | 0.576 | 0.093 | 0.223 | 0.567 | 0.500 | **0.032** | 0.164 |
| **DCD** | P81605 | 0.113 | 0.186 | 0.087 | 0.124 | 0.252 | 0.227 | **0.039** | 0.259 |
| **DDX54** | Q8TDD1 | 0.604 | 0.932 | 0.404 | **0.046** | 0.452 | 0.233 | 0.702 | 0.246 |
| **DNHD1** | Q96M86 | 0.985 | **0.001** | 0.620 | 0.855 | **0.005** | **0.006** | 0.723 | **0.028** |
| **DROSHA** | Q9NRR4 | 0.014 | 0.062 | 0.054 | **0.021** | 0.085 | 0.191 | **0.048** | 0.419 |
| **EDEM3** | Q9BZQ6 | 0.977 | **0.000** | 0.513 | 0.926 | **0.000** | **0.003** | 0.734 | **0.002** |
| **ENO1** | P06733 | 0.019 | 0.438 | **0.013** | 0.246 | 0.420 | 0.460 | 0.212 | 0.423 |
| **F2** | P00734 | 0.424 | 0.923 | 0.116 | 0.165 | 0.878 | 0.766 | **0.023** | 0.488 |
| **FGA** | P02671 | 0.137 | 0.470 | 0.133 | 0.179 | 0.631 | 0.335 | **0.034** | 0.573 |
| **FGB** | P02675;P01597 | 0.898 | 0.321 | 0.909 | 0.135 | 0.257 | **0.049** | 0.143 | 0.137 |
| **GAB2** | Q9UQC2 | 0.536 | 0.061 | 0.961 | 0.376 | **0.023** | **0.046** | 0.834 | 0.140 |
| **GAPDHS** | O14556 | 0.843 | **0.017** | 0.945 | 0.274 | 0.051 | 0.425 | 0.516 | 0.236 |
| **GRXCR2** | A6NFK2 | 0.016 | 0.108 | 0.057 | **0.046** | 0.124 | 0.112 | 0.204 | 0.095 |
| **GUCY1B2** | O75343 | 0.604 | 0.116 | 0.258 | 0.664 | 0.379 | **0.024** | 0.112 | 0.215 |

| Gene Names | PG Protein Accessions | Intercept | NTT | Group | sex | NTT X Group | NTT X Sex | Group X Sex | NTT X Group X sex |
|---|---|---|---|---|---|---|---|---|---|
| **HEPHL1** | Q6MZM0 | 0.923 | **0.015** | 0.610 | 0.371 | 0.633 | **0.015** | 0.472 | 0.611 |
| **HUWE1** | Q7Z6Z7 | 0.413 | 0.520 | 0.823 | 0.370 | 0.443 | 0.151 | 0.571 | **0.026** |
| **IGFALS** | P35858 | 0.068 | 0.739 | 0.099 | **0.048** | 0.632 | 0.532 | **0.026** | 0.451 |
| **IGHM** | P01871;P01773 | 0.132 | 0.255 | **0.007** | 0.270 | 0.829 | 0.269 | **0.035** | 0.495 |
| **IGHV1OR21-1** | A6NJS3 | 0.029 | 0.090 | 0.078 | 0.053 | **0.036** | 0.159 | 0.169 | 0.166 |
| **KIF14** | Q15058 | 0.613 | 0.973 | 0.564 | 0.920 | 0.246 | 0.138 | 0.948 | **0.024** |
| **KRT23** | Q9C075 | 0.088 | 0.457 | 0.076 | **0.021** | 0.546 | 0.268 | **0.023** | 0.444 |
| **KRT6B** | P04259 | 0.117 | 0.441 | **0.022** | 0.921 | 0.319 | 0.071 | 0.444 | 0.230 |
| **KRT77** | Q7Z794 | 0.247 | 0.795 | 0.106 | 0.172 | 0.966 | 0.375 | **0.035** | 0.537 |
| **KRT9** | P35527 | 0.416 | 0.782 | 0.135 | 0.259 | 0.869 | 0.549 | **0.029** | 0.687 |
| **LTN1** | O94822 | 0.101 | 0.619 | **0.024** | 0.125 | 0.641 | 0.414 | 0.096 | 0.661 |
| **LYVE1** | Q9Y5Y7 | 0.414 | 0.662 | 0.434 | 0.476 | 0.605 | **0.032** | 0.686 | **0.021** |
| **MED30** | Q96HR3 | 0.384 | **0.040** | 0.723 | 0.136 | **0.046** | **0.025** | 0.299 | **0.035** |
| **MLPH** | Q9BV36 | 0.089 | 0.053 | **0.030** | 0.175 | 0.094 | 0.144 | 0.064 | 0.249 |
| **MMP25** | Q9NPA2 | 0.658 | 0.183 | 0.395 | 0.583 | 0.077 | 0.086 | 0.465 | **0.047** |
| **MYO5B** | Q9ULV0 | 0.023 | 0.844 | **0.006** | 0.169 | 0.510 | 0.786 | 0.167 | 0.665 |
| **NKIRAS1** | Q9NYS0 | 0.013 | 0.371 | **0.004** | 0.055 | 0.847 | 0.861 | **0.050** | 0.373 |
| **NR0B1** | P51843 | 0.995 | 0.077 | 0.646 | 0.665 | **0.046** | 0.124 | 0.822 | 0.096 |
| **NRIP1** | P48552 | 0.344 | 0.877 | 0.075 | 0.831 | 0.613 | **0.039** | 0.171 | **0.040** |
| **NRXN1** | Q9ULB1 | 0.021 | 0.129 | **0.015** | **0.041** | 0.235 | 0.279 | 0.161 | 0.718 |
| **OSBPL1A** | Q9BXW6 | 0.200 | **0.004** | 0.273 | 0.170 | **0.014** | **0.012** | 0.224 | **0.035** |
| **PFN2** | P35080 | 0.084 | 0.699 | **0.038** | 0.400 | 0.611 | 0.256 | 0.138 | 0.112 |
| **PHACTR1** | Q9C0D0 | 0.148 | 0.800 | 0.075 | 0.058 | 0.948 | 0.886 | **0.022** | 0.840 |
| **PLG** | P00747 | 0.602 | 0.712 | 0.415 | 0.188 | 0.965 | 0.144 | **0.034** | 0.600 |
| **PRDX6** | P30041 | 0.612 | 0.072 | 0.836 | 0.955 | **0.017** | 0.179 | 0.628 | 0.153 |
| **QSOX1** | O00391 | 0.080 | 0.623 | 0.048 | 0.214 | 0.110 | 0.422 | 0.685 | **0.006** |
| **RBM44** | Q6ZP01 | 0.079 | 0.079 | 0.127 | 0.617 | **0.034** | 0.221 | 0.684 | 0.079 |
| **RELN** | P78509 | 0.430 | 0.987 | 0.552 | 0.691 | 0.727 | 0.200 | 0.488 | **0.047** |
| **RIC8B** | Q9NVN3 | 0.120 | **0.024** | **0.031** | 0.166 | **0.028** | 0.054 | **0.025** | **0.024** |
| **RPL14** | P50914 | 0.390 | 0.488 | 0.911 | **0.012** | 0.573 | 0.708 | 0.168 | 0.911 |
| **SERPINA1** | P01009 | 0.085 | 0.302 | 0.163 | 0.097 | 0.356 | 0.394 | **0.033** | 0.354 |
| **SERPINF1** | P36955 | 0.032 | 0.324 | **0.031** | **0.013** | 0.587 | 0.243 | **0.006** | 0.176 |
| **SKAP1** | Q86WV1 | 0.123 | 0.204 | **0.029** | **0.027** | 0.450 | 0.105 | **0.002** | 0.360 |
| **SLK** | Q9H2G2 | 0.561 | 0.428 | 0.741 | 0.820 | **0.037** | 0.324 | 0.740 | **0.009** |
| **SORCS2** | Q96PQ0 | 0.023 | 0.315 | **0.001** | 0.173 | 0.756 | 0.190 | **0.014** | 0.446 |
| **SOS2** | Q07890 | 0.645 | **0.018** | 0.755 | 0.913 | **0.020** | 0.113 | 0.315 | 0.177 |
| **SPARC** | P09486 | 0.074 | 0.869 | **0.044** | 0.183 | 0.510 | 0.996 | 0.151 | 0.667 |
| **SPTAN1** | Q13813 | 0.171 | 0.511 | 0.136 | **0.043** | 0.983 | 0.807 | **0.015** | 0.711 |
| **TEX11** | Q8IYF3 | 0.999 | 0.065 | 0.830 | 0.801 | 0.093 | 0.111 | 0.808 | **0.038** |
| **TGM1** | P22735 | 0.218 | **0.004** | 0.523 | **0.034** | **0.006** | 0.064 | 0.143 | 0.126 |
| **TGM3** | Q08188 | 0.053 | 0.187 | **0.025** | 0.111 | 0.370 | 0.279 | 0.080 | 0.656 |
| **TRAP1** | Q12931 | 0.039 | 0.056 | **0.013** | 0.187 | 0.307 | 0.454 | 0.097 | 0.969 |
| **TSC1** | Q92574 | 0.001 | 0.065 | **0.002** | **0.004** | 0.180 | 0.084 | **0.001** | 0.259 |
| **TTC36** | A6NLP5 | 0.039 | 0.393 | **0.022** | 0.179 | 0.412 | 0.670 | 0.176 | 0.867 |

| Gene Names | PG Protein Accessions | Intercept | NTT | Group | sex | NTT X Group | NTT X Sex | Group X Sex | NTT X Group X sex |
|---|---|---|---|---|---|---|---|---|---|
| **TXN** | P10599 | 0.111 | **0.001** | 0.126 | 0.243 | **0.006** | **0.003** | 0.595 | 0.133 |
| **ZG16B** | Q96DA0 | 0.255 | 0.673 | 0.074 | 0.193 | 0.834 | 0.605 | **0.035** | 0.663 |
| **ZNFX1** | Q9P2E3 | 0.448 | 0.989 | 0.254 | 0.817 | 0.484 | 0.109 | 0.223 | **0.028** |
| | P01597 | 0.048 | 0.079 | **0.048** | **0.048** | 0.347 | 0.271 | 0.088 | 0.859 |
| | P01772 | 0.771 | 0.136 | 0.730 | 0.751 | 0.076 | 0.122 | 0.899 | **0.033** |
| | P01778 | 0.389 | 0.235 | 0.377 | 0.577 | 0.158 | 0.094 | 0.473 | **0.049** |
| | P80422 | 0.395 | 0.786 | 0.327 | 0.111 | 0.506 | 0.756 | **0.031** | 0.864 |
| | P01619 | 0.942 | 0.944 | 0.942 | 0.230 | 0.946 | **0.042** | 0.312 | **0.042** |

Abbreviations: DAS-NTT, day-adjusted scores of normo-to-tachy ratio.
