# Supplementary material for: Molecular classification of the placebo effect in nausea
Source: PLoS One. 2020 Sep 23;15(9):e0238533. doi: 10.1371/journal.pone.0238533 (PMC7511022; doi:10.1371/journal.pone.0238533)
Supplement: S5 Table — (PDF) [file pone.0238533.s007.pdf]

**S5 Table: Enriched GO groups based on proteins for which a significant amount of variance could be explained by the factors 'group', 'sex', by 'DAS-Nausea', or by any of the interaction terms.**

| P-value | GO group | Gene Names |
|---|---|---|
| 0.000 | cholesterol metabolic process | APOL1 APOA2 APOB APP PON1 OSBPL1A |
| 0.000 | cell adhesion | COL6A5 FN1 APP GP1BA AZGP1 ACTN2 IGFALS PKP1 POSTN GRHL2 |
| 0.001 | protein localization to plasma membrane | ANXA2 FLNA ACTN2 SKAP1 |
| 0.002 | mating behavior | APP MAPK8IP2 |
| 0.004 | response to insulin | EIF6 EPM2AIP1 TSC1 |
| 0.005 | regulation of translation | APP TSC1 |
| 0.005 | fertilization | APOB TEX11 |
| 0.005 | positive regulation of stress-activated MAPK cascade | MAPK8IP2 CARD9 |
| 0.005 | activation of GTPase activity | SGSM1 TSC1 |
| 0.008 | neural tube closure | GRHL2 TSC1 FZD3 |
| 0.009 | regulation of protein phosphorylation | FN1 HSPB1 |
| 0.009 | hair follicle development | FZD3 KRT84 |
| 0.009 | protein O-linked glycosylation | PLOD2 POMT2 |
| 0.009 | positive regulation of peptidase activity | FN1 APP |
| 0.009 | cell junction assembly | FLNA GRHL2 |
| 0.009 | sodium ion transmembrane transport | GRIK4 SCN2A |
| 0.009 | synapse organization | APP TSC1 |
| 0.009 | regulation of NMDA receptor activity | APP MAPK8IP2 |
| 0.013 | post-translational protein modification | APOL1 SERPINC1 APOA2 FN1 APOB APP |
| 0.015 | adult locomotory behavior | APP TSC1 |
| 0.015 | social behavior | MAPK8IP2 NRXN1 |
| 0.015 | ionotropic glutamate receptor signaling pathway | APP GRIK4 |
| 0.015 | positive regulation of JNK cascade | APP CARD9 |
| 0.015 | neuron apoptotic process | APP SCN2A |
| 0.015 | excitatory postsynaptic potential | MAPK8IP2 GRIK4 |
| 0.015 | cellular response to retinoic acid | KRT13 SERPINF1 |
| 0.017 | cytoskeleton organization | KRT6B KRT16 KRT13 KRT84 |
| 0.020 | cornification | KRT6B KRT16 KRT13 FLG PKP1 KRT84 |
| 0.021 | negative regulation of protein kinase activity | HSPB1 GP1BA |
| 0.021 | myelination | TSC1 SCN2A |
| 0.023 | cellular protein metabolic process | APOL1 SERPINC1 APOA2 FN1 APOB APP IGFALS |
| 0.029 | positive regulation of gene expression | FN1 APOB APP PKP1 |
| 0.029 | I-kappaB kinase/NF-kappaB signaling | CARD9 NKIRAS1 |
| 0.029 | learning | APP NRXN1 |
| 0.029 | chylomicron remodeling | APOA2 APOB |
| 0.029 | very-low-density lipoprotein particle assembly | ACSL3 APOB |
| 0.029 | negative regulation of catalytic activity | ANXA2 PHACTR1 |

| P-value | GO group | Gene Names |
|---|---|---|
| 0.029 | establishment of skin barrier | KRT16 FLG |
| 0.030 | platelet aggregation | HSPB1 GP1BA FLNA |
| 0.033 | keratinization | KRT6B KRT16 KRT13 PKP1 KRT84 |
| 0.037 | kidney development | SERPINF1 TSC1 |
| 0.037 | low-density lipoprotein particle remodeling | APOA2 APOB |
| 0.037 | chylomicron assembly | APOA2 APOB |
| 0.037 | neuromuscular process controlling balance | APP NRXN1 |
| 0.045 | vesicle-mediated transport | OSBPL1A MYO5B |
| 0.045 | actin cytoskeleton reorganization | FLNA PHACTR1 |
| 0.045 | positive regulation of fibroblast proliferation | FN1 ANXA2 |
| 0.045 | protein heterooligomerization | FGFRL1 TSC1 |
| 0.048 | retina homeostasis | HSPB1 AZGP1 ZG16B |

Note: Significant GO groups with single genes were omitted.
Abbreviations: DAS-Nausea, day-adjusted scores of nausea.
