# Supplementary material for: Molecular classification of the placebo effect in nausea
Source: PLoS One. 2020 Sep 23;15(9):e0238533. doi: 10.1371/journal.pone.0238533 (PMC7511022; doi:10.1371/journal.pone.0238533)
Supplement: S6 Table — (PDF) [file pone.0238533.s008.pdf]

S6 Table: Enriched groups of proteins for which a significant amount of variance could be explained by the factors 'group', 'sex', by 'DAS-MS', or by any of the interaction terms.

P-value	GO group	Gene Names
0.002	adult behavior	NRXN1 SHANK1
0.002	vocal learning	HTT NRXN1
0.002	negative regulation of catalytic activity	ANXA2 RNH1 PHACTR1
0.004	vesicle-mediated transport	OSBPL1A KIF17 MYO5B
0.004	neutrophil degranulation	CEP290 CFD A1BG GSN S100A9 ANXA2 S100A7 ABCA13 SERPINB12 BIN2
0.004	protein destabilization	GSN HTT
0.004	vocalization behavior	NRXN1 SHANK1
0.004	amyloid fibril formation	GSN MAPT
0.009	positive regulation of neurogenesis	SERPINF1 SPEN
0.009	interaction with symbiont	PLG DDB1
0.009	positive regulation of excitatory postsynaptic potential	NRXN1 SHANK1
0.012	transcription, DNA-templated	ENO1 TXN NRIP1 ZNF438 DDX54 SPEN BRMS1
0.014	activation of cysteine-type endopeptidase activity involved in apoptotic process	S100A9 MAPT
0.014	social behavior	NRXN1 SHANK1
0.014	tissue regeneration	PLG GSN
0.014	cellular response to retinoic acid	KRT13 SERPINF1
0.014	negative regulation of gene expression	MAPT SERPINF1 SLC35C2
0.017	fibrinolysis	PLG HRG ANXA2
0.017	antimicrobial humoral immune response mediated by antimicrobial peptide	HRG S100A9 S100A7
0.018	negative regulation of transcription from RNA polymerase II promoter	ENO1 TXN NRIP1 SPEN BRMS1
0.020	response to radiation	TXN KRT13
0.027	negative regulation of fibrinolysis	PLG HRG
0.030	positive regulation of transcription, DNA-templated	CEP290 SPP1 NRIP1 SKAP1
0.031	DNA repair	ATR DDB1 POLI
0.034	RNA processing	DDX54 ADAD1
0.034	peptidyl-serine phosphorylation	ATR RICTOR
0.034	negative regulation of cell growth	HRG ENO1
0.034	neuromuscular process controlling balance	NRXN1 SHANK1
0.042	regulation of gene expression	HRG RICTOR
0.042	actin cytoskeleton reorganization	RICTOR PHACTR1
0.044	aging	GSN VCAM1 SERPINF1
0.048	extracellular matrix disassembly	PLG GSN SPP1

Note: Significant GO groups with single genes were omitted.
Abbreviations: DAS-MS, day-adjusted scores of motion sickness.
