# Supplementary material for: Molecular classification of the placebo effect in nausea
Source: PLoS One. 2020 Sep 23;15(9):e0238533. doi: 10.1371/journal.pone.0238533 (PMC7511022; doi:10.1371/journal.pone.0238533)
Supplement: S7 Table — (PDF) [file pone.0238533.s009.pdf]

S7 Table: Enriched groups of proteins for which a significant amount of variance could be explained by the factors 'group', 'sex', by 'DAS-NTT, or by any of the interaction terms.

P-value	GO group	Gene Names
0.001	positive regulation of peptidyl-serine phosphorylation	APP TXN CD44 PFN2
0.002	regulation of grooming behavior	CNTNAP4 NRXN1
0.002	copper ion transport	CP HEPHL1
0.002	positive regulation of small GTPase mediated signal transduction	RELN SOS2
0.002	positive regulation of synaptic transmission, glutamatergic	RELN NRXN1
0.002	positive regulation of synapse maturation	RELN NRXN1
0.002	postsynaptic density protein 95 clustering	RELN NRXN1
0.002	hippocampus development	RELN KIF14 TSC1
0.003	axon guidance	RELN SPTAN1 NRXN1 GAB2
0.004	kidney development	SERPINF1 TSC1 C5orf42
0.005	cell redox homeostasis	QSOX1 TXN PRDX6
0.006	regulation of translation	APP TSC1
0.006	dendrite development	APP RELN
0.006	cell envelope organization	TGM1 TGM3
0.006	positive regulation of long-term synaptic potentiation	APP RELN
0.011	potassium ion transport	ABCC9 TSC1
0.011	response to lead ion	APP SPARC
0.011	negative regulation of macroautophagy	QSOX1 TSC1
0.011	positive regulation of heterotypic cell-cell adhesion	FGA CD44
0.011	synapse organization	APP TSC1
0.011	bone development	SPARC ANKRD11
0.011	regulation of NMDA receptor activity	APP RELN
0.011	positive regulation of excitatory postsynaptic potential	RELN NRXN1
0.012	cellular protein metabolic process	QSOX1 CP F2 PLG SERPINA1 FGA APP IGFALS
0.016	platelet degranulation	QSOX1 PLG SERPINA1 FGA APP SPARC ACTN2
0.017	adult locomotory behavior	APP TSC1
0.017	hyaluronan catabolic process	CD44 LYVE1
0.017	androgen receptor signaling pathway	NRIP1 MED30
0.018	cell-matrix adhesion	FGA CD44 TSC1 LYVE1
0.019	cellular oxidant detoxification	APOM TXN PRDX6
0.021	neutrophil degranulation	QSOX1 SERPINA1 CD44 PRDX6 SPTAN1 HUWE1 ABCA13 MMP25 BIN2
0.022	cell adhesion	APP COL6A3 AZGP1 ACTN2 IGFALS RELN CNTNAP4
0.022	fibrinolysis	F2 PLG FGA
0.023	transmembrane transport	ABCC9 AZGP1 ABCA13 ABCA6

0.024	transcription initiation from RNA polymerase II promoter	NR0B1 MED30
0.024	activation of protein kinase activity	KIF14 SLK
0.024	regulation of molecular function	ABCC9 NRXN1
0.031	lipid transport	APOC4 ABCA13 ABCA6
0.031	protein stabilization	CPN2 PFN2 TSC1
0.032	gluconeogenesis	GAPDHS ENO1
0.032	learning	APP NRXN1
0.032	negative regulation of fibrinolysis	F2 PLG
0.033	oxidation-reduction process	QSOX1 CP TXN PRDX6 HEPHL1
0.035	extracellular matrix organization	FGA APP SPARC COL6A3 CD44
0.040	keratinization	KRT6B KRT9 TGM3 KRT77 KRT23
0.041	positive regulation of transcription, DNA-templated	NRIP1 ATAD2 SKAP1 MED30
0.041	cellular protein modification process	TGM1 TGM3
0.041	neuromuscular process controlling balance	APP NRXN1
0.048	post-translational protein modification	QSOX1 CP SERPINA1 FGA APP

Note: Significant GO groups with single genes were omitted.
Abbreviations: DAS-NTT, day-adjusted scores of normo-to-tachy ratio.
