# Supplementary material for: Molecular classification of the placebo effect in nausea
Source: PLoS One. 2020 Sep 23;15(9):e0238533. doi: 10.1371/journal.pone.0238533 (PMC7511022; doi:10.1371/journal.pone.0238533)
Supplement: S8 Table — Retrieved from analyses of covariance (ANCOVA) on vection-induced fold changes of proteins on Day 2 (between-subject factors ‘group’ and ‘sex’, covariate fold changes on Day 1). (PDF) [file pone.0238533.s010.pdf]

S8 Table: 74 proteins that were differentially regulated in the placebo group as compared to the control group. Retrieved from analyses of covariance (ANCOVA) on vection-induced fold changes of proteins (PFC) on Day 2 (between-subject factors 'group' and 'sex', covariate PFC on Day 1).

Gene Names	Protein Accessions	*P*-value (Group)	No. of Peptides	Mean PFC day 1 (All)	Mean PFC day2 (Control)	Mean PFC day2 (Placebo)
**A2M**	P01023	0.000	633	0.100	0.052	-0.033
**ABCC9**	O60706	0.002	1	0.150	-1.452	0.286
**AHSG**	P02765	0.013	70	0.072	-0.145	0.069
**ALB;C3;APOA2**	P02768;P01024; P01781;P02652	0.035	1	0.579	0.833	-0.433
**ANKRD23**	Q86SG2	0.041	1	0.928	-0.863	0.169
**APOA1**	P02647	0.002	193	0.000	-0.084	0.053
**APOB**	P04114	0.000	1102	0.005	0.057	-0.037
**C19orf68**	Q86XI8	0.015	2	0.685	-0.364	0.096
**C1QC**	P02747	0.031	20	0.240	0.142	-0.060
**C3**	P01024	0.000	828	0.103	0.064	-0.038
**C4A**	P0C0L4	0.000	411	0.922	0.113	-0.017
**C4BPB**	P20851	0.044	6	0.837	0.324	-0.272
**C5**	P01031	0.020	158	0.178	0.106	-0.020
**CAT**	P04040	0.040	7	0.514	0.719	-0.057
**CCSER1**	Q9C0I3	0.028	1	0.975	-0.252	0.222
**CD163**	Q86VB7	0.006	2	0.077	1.145	0.019
**CFH**	P08603	0.040	135	0.014	0.104	-0.012
**COG5**	Q9UP83	0.048	3	0.284	-0.537	0.270
**CP**	P00450	0.001	342	0.004	0.092	-0.040
**CRB1**	P82279	0.041	3	0.766	-0.411	0.108
**EVC**	P57679	0.020	1	0.003	-0.738	0.698
**F10**	P00742	0.003	15	0.954	-0.415	0.190
**FGA**	P02671	0.001	391	0.000	0.128	-0.011
**FGB**	P02675	0.000	313	0.030	0.048	-0.086
**FGG**	P02679	0.000	250	0.025	0.089	-0.080
**GAPDHS**	O14556	0.006	1	0.657	-2.004	2.724
**GC**	P02774	0.008	111	0.569	0.210	0.020
**GYLTL1B**	Q8N3Y3	0.041	1	0.822	-0.781	0.218
**HBA1**	P69905	0.000	64	0.316	0.042	-0.397
**HBB**	P68871	0.001	47	0.005	-0.071	-0.357
**HNRNPA1L2**	Q32P51	0.022	2	0.637	1.500	-0.342
**HP**	P00738	0.036	122	0.415	0.034	-0.089
**HPR;HP;GPLD1;FGB;GC;MAPT**	P00739;P00738; P80108;P02675; P02774;P10636	0.015	1	0.689	0.827	-0.214
**HPS3**	Q969F9	0.009	1	0.001	-1.027	0.914
**HSPA8**	P11142	0.030	10	0.821	0.472	-0.311
**IGFALS**	P35858	0.046	33	0.828	0.192	-0.053
**IGHG2**	P01859	0.032	85	0.043	0.120	-0.032
**IGHG3**	P01860	0.000	48	0.115	0.321	-0.152

**IGHV3-23**	P01764	0.010	24	0.640	0.204	-0.123
**IGLC7**	A0M8Q6	0.029	4	0.404	-0.558	0.306
**JUP**	P14923	0.042	17	0.589	0.552	-0.104
**KIAA1614**	Q5VZ46	0.001	1	0.063	-1.154	0.684
**KRT10**	P13645	0.001	121	0.000	-0.058	0.143
**KRT6B;FGB**	P04259;P02675	0.013	1	0.955	0.576	-0.246
**LTBP1**	Q14766	0.042	4	0.102	-0.189	0.226
**LYZ**	P61626	0.044	7	0.696	-0.381	0.377
**MAPK8IP2**	Q13387	0.037	2	0.737	-0.356	0.110
**MYO18B**	Q8IUG5	0.005	1	0.645	-0.417	0.959
**MYO5B**	Q9ULV0	0.042	2	0.998	0.056	-0.629
**NKIRAS1**	Q9NYS0	0.016	1	0.137	-1.563	1.074
**ORM1**	P02763	0.016	38	0.878	-0.182	0.110
**PAPLN**	O95428	0.003	1	0.375	0.324	-0.648
**PDLIM1**	O00151	0.021	1	0.889	0.890	-1.432
**PFN2**	P35080	0.040	1	0.300	1.283	-0.719
**PIH1D2**	Q8WWB5	0.028	1	0.298	1.795	0.038
**PPIA**	P62937	0.048	4	0.953	0.635	-0.061
**PRDX2**	P32119	0.033	11	0.037	-0.025	-0.515
**PROC**	P04070	0.033	9	0.821	-0.406	0.359
**PRSS27**	Q9BQR3	0.028	2	0.062	-0.907	-0.117
**PZP**	P20742	0.042	24	0.013	0.295	-0.084
**RAB3D**	O95716	0.021	1	0.243	-0.899	0.121
**RPL14**	P50914	0.022	1	0.162	-2.392	0.323
**SERPINB12**	Q96P63	0.024	5	0.118	-0.296	0.370
**SERPIND1**	P05546	0.000	83	0.717	0.159	-0.118
**SPG11**	Q96JI7	0.027	1	0.501	0.551	-0.558
**TF**	P02787	0.012	355	0.000	0.079	-0.021
**TSPYL1**	Q9H0U9	0.039	2	0.854	-1.026	0.285
**TTC36**	A6NLP5	0.004	2	0.384	1.557	-0.521
**VIL1**	P09327	0.046	1	0.389	-0.107	0.870
**VWA3A**	A6NCI4	0.045	1	0.498	-0.453	0.476
N/A	P01703	0.006	4	0.040	-0.582	0.195
N/A	P01607	0.033	3	0.725	-0.369	0.416
N/A	P01614	0.034	2	0.406	-0.459	0.155
N/A	P06888	0.040	5	0.805	-0.509	0.224
