# Supplementary material for: Molecular classification of the placebo effect in nausea
Source: PLoS One. 2020 Sep 23;15(9):e0238533. doi: 10.1371/journal.pone.0238533 (PMC7511022; doi:10.1371/journal.pone.0238533)
Supplement: S9 Table — For list of 74 proteins, see S8 Table. (PDF) [file pone.0238533.s011.pdf]

S9 Table: Significant Gene enrichment (GO) terms and related proteins resulting from GO enrichment analyses on 74 proteins differentially regulated in the placebo and control groups. For list of 74 proteins, see S8 Table.

Gene enrichment (GO) terms	*P*-value (FDR-corr.)	Gene Names
platelet degranulation	0.022	A2M, APOA1, FGA, FGB, FGG, ORM1, AHSG, TF
positive regulation of substrate adhesion-dependent cell spreading	0.008	APOA1, FGA, FGB, FGG
vitamin transport	0.047	APOA1, GC
positive regulation of lipid storage	0.022	C3, APOB
hydrogen peroxide catabolic process	0.010	CAT, PRDX2, HBB, HBA1
cellular protein metabolic process	0.000	CP, C3, APOA1, FGA, FGG, AHSG, TF, PROC, APOB, SERPIND1, C4A, IGFALS, LYZ, LTBP1
post-translational protein modification	0.000	CP, C3, APOA1, FGA, FGG, AHSG, TF, PROC, APOB, SERPIND1, C4A, LTBP1
blood coagulation, common pathway	0.022	F10, FGA
blood coagulation	0.002	F10, FGA, FGB, FGG, PROC, SERPIND1, C4BPB, HBB
induction of bacterial agglutination	0.022	FGA, FGB
positive regulation of peptide hormone secretion	0.002	FGA, FGB, FGG
cellular protein complex assembly	0.002	FGA, FGB, FGG
negative regulation of extrinsic apoptotic signaling pathway via death domain receptors	0.005	FGA, FGB, FGG
positive regulation of heterotypic cell-cell adhesion	0.005	FGA, FGB, FGG
positive regulation of vasoconstriction	0.005	FGA, FGB, FGG
blood coagulation, fibrin clot formation	0.010	FGA, FGB, FGG
positive regulation of exocytosis	0.010	FGA, FGB, FGG
protein polymerization	0.010	FGA, FGB, FGG
plasminogen activation	0.022	FGA, FGB, FGG
negative regulation of endothelial cell apoptotic process	0.039	FGA, FGB, FGG
toll-like receptor signaling pathway	0.010	FGA, FGB, FGG, APOB
platelet aggregation	0.038	FGA, FGB, FGG, HBB
positive regulation of protein secretion	0.010	FGA, FGB, FGG, PPIA
response to hydrogen peroxide	0.004	HP, CAT, HBB, HBA1
cellular oxidant detoxification	0.022	HP, CAT, HBB, HBA1
positive regulation of cell death	0.018	HP, HBB, HBA1
acute-phase response	0.038	HP, ORM1, AHSG, CD163
regulation of complement activation	0.011	IGLC7, C3, C5, IGHV3-23, IGHG2, IGHG3, C1QC, CFH, C4A, C4BPB
complement activation	0.022	IGLC7, C3, C5, IGHV3-23, IGHG2, IGHG3, C1QC, CFH, C4A
innate immune response	0.047	IGLC7, IGHV3-23, IGHG2, IGHG3, FGA, FGB, C1QC, C4A, C4BPB
protein heterooligomerization	0.039	JUP, HBB, HBA1
negative regulation of endopeptidase activity	0.001	PAPLN, A2M, C3, C5, AHSG, SERPIND1, C4A, PZP, SERPINB12
neutrophil degranulation	0.005	RAB3D, HP, C3, ORM1, AHSG, CAT, HSPA8, JUP, LYZ, PPIA, HBB, SERPINB12
