# Supplementary material for: Molecular classification of the placebo effect in nausea
Source: PLoS One. 2020 Sep 23;15(9):e0238533. doi: 10.1371/journal.pone.0238533 (PMC7511022; doi:10.1371/journal.pone.0238533)
Supplement: S10 Table — For GO terms, see S9 Table. (PDF) [file pone.0238533.s012.pdf]

S10 Table: Significant prediction of DAS-Nausea, DAS-MS, and DAS-NTT in the placebo and control groups by protein fold changes of significantly enriched GO terms. For GO terms, see S9 Table.

Gene enrichment (GO) term	DAS-Nau Ctrl	DAS-MS Ctrl	DAS-NTT Ctrl	DAS-Nau Plc	DAS-MS Plc	DAS-NTT Plc	Proteins
	***P*-value (FDR-corrected)**						
**Placebo group**							
acute-phase response	0.140	0.781	0.336	**0.003**	0.771	0.297	HP, ORM1, AHSG, CD163
blood coagulation, common pathway	0.103	0.560	0.400	0.807	0.331	**0.023**	FGA
induction of bacterial agglutination	0.178	0.515	0.268	0.943	0.321	**0.015**	FGA, FGB
positive regulation of peptide hormone secretion	0.241	0.665	0.370	0.896	0.312	**0.038**	FGA, FGB, FGG
platelet aggregation	0.308	0.821	0.383	0.710	0.238	**0.020**	FGA, FGB, FGG, HBB
blood coagulation	0.445	0.226	0.533	0.598	0.360	**0.050**	FGA, FGB, FGG, SERPIND1, HBB
complement activation	0.063	0.164	0.804	0.706	0.698	**0.026**	C3, C5, IGHV3_23, IGHG2, IGHG3, C1QC, CFH, C4A, IGLC7
**Control group**							
positive regulation of substrate adhesion-dependent cell spreading	0.413	**0.000**	0.492	0.952	0.706	**0.019**	APOA1, FGA, FGB, FGG
protein heterooligomerization	**0.049**	**0.006**	0.540	0.404	0.095	0.072	HBB, HBA1
positive regulation of cell death	0.110	**0.021**	0.714	0.632	0.182	0.130	HP, HBB, HBA1
hydrogen peroxide catabolic process	0.087	**0.022**	0.204	0.492	0.184	0.141	CAT, HBB, HBA1

Abbreviations: DAS, day-adjusted scores; Nau, nausea; Ctrl, control group; MS, motion sickness score; NTT, normo-to-tachy-ratio; Plc, placebo group.
