# Supplementary material for: Molecular classification of the placebo effect in nausea
Source: PLoS One. 2020 Sep 23;15(9):e0238533. doi: 10.1371/journal.pone.0238533 (PMC7511022; doi:10.1371/journal.pone.0238533)
Supplement: S11 Table — (PDF) [file pone.0238533.s013.pdf]

S11 Table: Proteins at baseline of Day 2 differentiating between placebo responders (≥50% reduction in nausea) and placebo non-responders according to ROC curves.

Gene Names	Protein Accessions	Protein Descriptions	P-value
ACTN2	P35609	Alpha-actinin-2	0.003
MRPL15	Q9P015	39S ribosomal protein L15. mitochondrial	0.008
GAPDHS	O14556	Glyceraldehyde-3-phosphate dehydrogenase. testis-specific	0.010
KRT31	Q15323	Keratin. type I cuticular Ha1	0.011
ACAN	P16112	Aggrecan core protein	0.012
IGHM;	P01871;P01773	Ig mu chain C region;Ig heavy chain V-III region BUR	0.014
TFRC	P02786	Transferrin receptor protein 1	0.264
NUCB1	Q02818	Nucleobindin-1	0.296
FAM83G	A6ND36	Protein FAM83G	0.353
FER1L5	A0AVI2	Fer-1-like protein 5	0.417
SLC9A3R1	O14745	Na(+)/H(+) exchange regulatory cofactor NHE-RF1	0.782

Abbreviation: ROC, receiver operating characteristics.
