# Supplementary material for: Molecular classification of the placebo effect in nausea
Source: PLoS One. 2020 Sep 23;15(9):e0238533. doi: 10.1371/journal.pone.0238533 (PMC7511022; doi:10.1371/journal.pone.0238533)
Supplement: S12 Table — (PDF) [file pone.0238533.s014.pdf]

S12 Table: Protein signature at baseline of Day 2 differentiating between placebo responders (≥50% reduction in Motion sickness) and placebo non-responders according to ROC curves.

Gene Names	Protein Accessions	Protein Descriptions	P-value
IGKV1D-16	P01601	Ig kappa chain V-ID region 16 (Fragment)	0.004
IGHV3-23	P01764	Ig heavy chain V-III region 23	0.010
ARHGDIB	P52566	Rho GDP-dissociation inhibitor 2	0.012
WDR62	O43379	WD repeat-containing protein 62	0.015
MASP2	O00187	Mannan-binding lectin serine protease 2	0.017
NINL	Q9Y2I6	Ninein-like protein	0.034
	P01597	Ig kappa chain V-I region DEE	0.055
ADCY2	Q08462	Adenylate cyclase type 2	0.072
GZMB	P10144	Granzyme B	0.110
QSOX1	O00391	Sulfhydryl oxidase 1	0.174
ZNF806	P0C7X5	Zinc finger protein 806	0.227
IGHG1	P01857	Ig gamma-1 chain C region	0.417
LRP5	O75197	Low-density lipoprotein receptor-related protein 5	0.544
TXN	P10599	Thioredoxin	0.647
MCM3AP	O60318	Germinal-center associated nuclear protein	0.789
CP	P00450	Ceruloplasmin	0.824
PPIP5K2	O43314	Inositol hexakisphosphate and diphosphoinositol-pentakisphosphate kinase 2	0.843

Abbreviation: ROC, receiver operating characteristics.
